# High-accuracy current generation in the nanoampere regime from a silicon single-trap electron pump

**DOI:** 10.1038/srep45137

**Published:** 2017-03-21

**Authors:** Gento Yamahata, Stephen P. Giblin, Masaya Kataoka, Takeshi Karasawa, Akira Fujiwara

**Affiliations:** 1 NTT Basic Research Laboratories, NTT Corporation, 3-1 Morinosato Wakamiya, Atsugi, Kanagawa 243-0198, Japan; 2 National Physical Laboratory, Hampton Road, Teddington, Middlesex TW11 0LW, United Kingdom

## Abstract

A gigahertz single-electron (SE) pump with a semiconductor charge island is promising for a future quantum current standard. However, high-accuracy current in the nanoampere regime is still difficult to achieve because the performance of SE pumps tends to degrade significantly at frequencies exceeding 1 GHz. Here, we demonstrate robust SE pumping via a single-trap level in silicon up to 7.4 GHz, at which the pumping current exceeds 1 nA. An accuracy test with an uncertainty of about one part per million (ppm) reveals that the pumping current deviates from the ideal value by only about 20 ppm at the flattest part of the current plateau. This value is two orders of magnitude better than the best one reported in the nanoampere regime. In addition, the pumping accuracy is almost unchanged up to 7.4 GHz, probably due to strong electron confinement in the trap. These results indicate that trap-mediated SE pumping is promising for achieving the practical operation of the quantum current standard.

Electron transport through a nanowire, a quantum dot, or a localized level has been intensively studied for application to atomic-scale electronic devices^1^,^2^, quantum information processing^3^,^4^, and a metrological current standard^5^. One of the important techniques for these applications is fast and precise manipulation of electrons. A clock-controlled single-electron (SE) pump is a promising device for achieving accurate manipulation of a single electron, which can generate quantized electric current *ef*, where *e* is the elementary charge and *f* is the input clock frequency. With increasing frequency, we can obtain a high pumping current level. An ability to generate a wide range of current is crucial for realizing an SE-pumping-based quantum current standard^5^, which could directly realize the new SI ampere^6^. In addition, a higher current is necessary in order to resolve the current quantization to a higher level of accuracy. Therefore, high-frequency operation of the SE pump is important.

A valuable application of the current standard is the quantum metrology triangle (QMT) experiment^7^,^8^,^9^,^10^, in which a current generated from the SE-pumping-based current standard is compared with that generated from a combination of the quantum Hall resistance standard and Josephson voltage standard. From this experiment, we can check the consistency of the fundamental physical constants. To close the QMT with a higher accuracy [for example, better than 0.1 parts per million (ppm)] using a direct current comparison^10^, a high current level as well as a high accuracy in the SE pump is practically necessary because, otherwise, the time required to attain a sub-ppm random uncertainty would become unreasonably long. For this purpose, it would be desirable to generate a current of more than 1 nA, corresponding to a frequency higher than about 6.3 GHz.

A promising candidate for a high-speed SE pump in the gigahertz regime is a tunable-barrier SE pump with a semiconductor charge island^11^,^12^,^13^,^14^,^15^,^16^. Recently, several high-accuracy current measurements using tunable-barrier SE pumps, in which SEs are transferred via an electrically defined charge island, have been performed for GaAs^17^,^18^,^19^ and Si^20^. In these measurements, ppm or sub-ppm quantization accuracy at up to 1 GHz has been demonstrated. However, the accuracy of this kind of SE pump significantly degrades with increasing frequency^15^,^17^,^19^,^21^; in earlier work, we demonstrated the highest-speed operation at 6.5 GHz but with accuracy limited to about 1000 ppm^20^. One possible cause of the degradation is that the charging energy relevant to determining the charge quanization accuracy is frequency dependent^15^. Another possibility is nonadiabatic excitation^22^.

One approach to enhance the charging energy or suppress nonadiabatic excitation is to use SE pumping via a localized state such as single-trap^23^,^24^ or single-dopant level^25^,^26^,^27^,^28^,^29^. We have recently reported a clear current plateau at 3.5 GHz by pumping SEs via a single-trap level (single-trap electron pump)^24^, which implies the possibility of high-speed operation. However, the accuracy of single-trap electron pumps has not been precisely evaluated, and there have been no reports of nanoampere-level pumping current.

In this letter, we report high-speed operation of a single-trap electron pump at up to 7.4 GHz (*ef* ~ 1.19 nA). The pumping current is evaluated using a high-accuracy measurement setup based on a precisely calibrated standard resistor with 0.8-ppm uncertainty. At 7.4 GHz, the flattest part of the one-electron plateau is offset from *ef* by only about 20 ppm. At such a high frequency, this represents an improvement in pumping accuracy of two orders of magnitude compared to a previous report^20^.

## Results

### Device structure and operating principle

**Figure 1:**
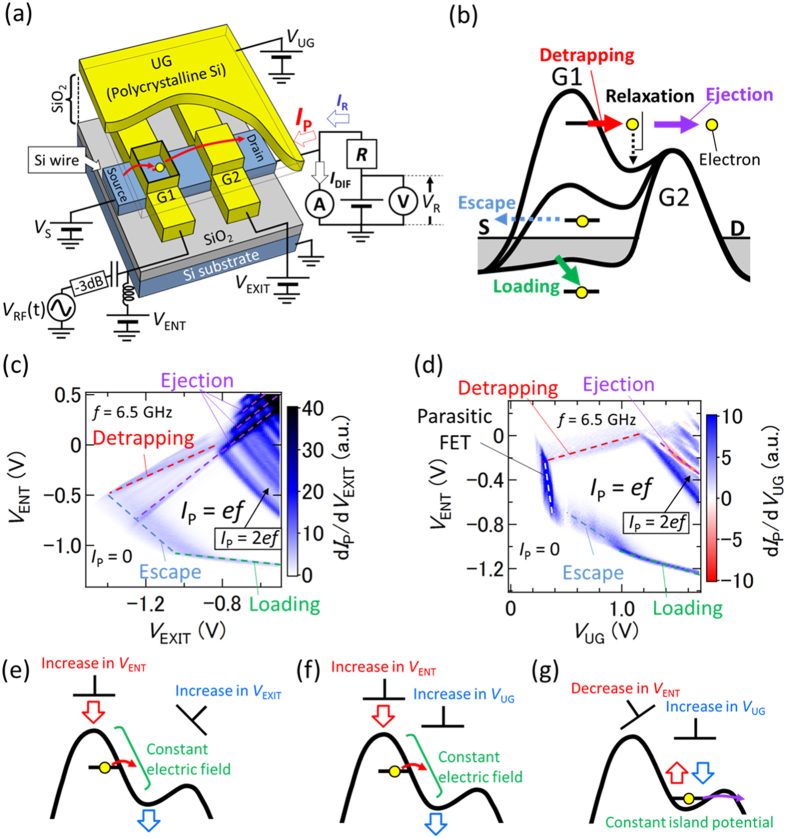
(**a**) Schematic of the device structure with electrical connections. The trap should be located under the right edge of G1. *R* is an 1-GΩ standard resistor calibrated against the quantum Hall resistance standard. Voltage applied to *R* is measured using a voltmeter calibrated against the Josephson voltage standard. *V*_R_ is the readout of the voltmeter. *I*_R_ = *V*_R_/*R* is a reference current, and *I*_DIF_ = *I*_R_ − *I*_P_ is the current difference between *I*_R_ and the pumping current *I*_P_. (**b**) Electron potential diagram during the SE pumping via a single-trap level. S and D are the source and drain leads, respectively. (**c**) First derivative of *I*_P_ with respect to *V*_EXIT_ as a function of *V*_EXIT_ and *V*_ENT_ at 6.5 GHz, where *V*_UG_ = 1.3 V, *V*_S_ = 0 V, and *P* = 14 dBm. (**d**) First derivative of *I*_P_ with respect to *V*_UG_ as a function of *V*_UG_ and *V*_ENT_ at 6.5 GHz, where *V*_EXIT_ = −0.8 V, *V*_S_ = 0 V, and the power of the high-frequency signal *P* = 14 dBm. (**e**,**f**) Potential diagrams during the detrapping process. Increasing *V*_ENT_ lowers the potential of the entrance barrier (red arrows) and increasing both *V*_EXIT_ and *V*_UG_ lowers the island potential (blue arrows). Therefore, these voltage applications keep the electric field at the trap level constant. Note that the modulation of the exit barrier by *V*_EXIT_ is not related to the detrapping process. (**g**) Potential diagram during the ejection process. Decreasing *V*_ENT_ raises (red arrow) the island potential; increasing *V*_UG_ lowers (blue arrow) it. This keeps the island potential constant, leading to a constant ejection probability. Note that the modulation of the entrance barrier by *V*_ENT_ is not related to the ejection process.

We fabricate a device with a double-layer gate structure on a Si wire [Fig. 1(a)] (see Methods). The upper gate is used to induce charge carriers in the Si wire by applying a voltage *V*_UG_. Entrance and exit potential barriers are formed in the Si wire by applying DC voltages to G1 and G2 (*V*_ENT_ and *V*_EXIT_), respectively. In addition, a high-frequency sinusoidal signal with frequency *f* and power *P* is added to G1. A DC voltage *V*_S_ is applied to the source lead to check its dependence of the pumping characteristics. The device is characterized using the high-accuracy current measurement system (see Methods). Here, we use one single-trap electron pump^24^. The origin of the trap level is probably an interface trap between Si and SiO_2_.

Figure 1(b) shows a schematic of an electron potential diagram during SE pumping via a single-trap level. When the height of the entrance barrier is low, an electron is loaded into the trap level. As the entrance barrier rises, the electron could escape to the source lead, causing current quantization error. This process is similar to the nonequilibrium SE capture in a charge island as the entrance barrier rises^30^. Then, the electron is detrapped to the island, followed by its ejection over the exit barrier to the drain lead. When one electron is transferred in each cycle, output current *I*_P_ = *ef*. To achieve a low escape probability and a high detrapping probability, leading to high quantization accuracy, a single-trap level should be located under the right edge of the entrance barrier. Note that when the electron is on the island after it has been detrapped, it can be relaxed to the ground state in the island. This leads to a current decrease when the ejection from the ground state to the drain is slow. In addition to this trap-mediated pumping, electrons can be transferred via the charge island^12^,^20^, although the accuracy is limited to about 1000 ppm at 1 GHz in this specific device. In order to tune the number of transferred electrons in each cycle, it is necessary to modulate the trap level and island potential by changing voltages such as *V*_UG_, *V*_ENT_, and *V*_EXIT_.

### Mechanism of trap-mediated pumping

Figure 1(c) shows d*I*_P_/d*V*_EXIT_ as a function of *V*_EXIT_ and *V*_ENT_ at 6.5 GHz. Here, instead of using the high-accuracy measurement setup, we just use an ammeter to measure *I*_P_ (*V*_R_ ~ 0). In this map, there are boundaries determined by loading (green dashed line), escape (blue dashed line), and ejection (purple dashed lines), which are similar to those in a typical map of a tunable-barrier pump with a charge island^16^,^31^. In addition, we observe another boundary (red dashed line), which should correspond to the detrapping process. The detrapping boundary has a positive slope on the map, because, when *V*_EXIT_ is increased, *V*_ENT_ also needs to be increased to maintain the electric field across the trap level [Fig. 1(e)], which affects the detrapping probability. However, the slope is smaller than that of the boundaries determined by the ejection. This is because the trap is further away from G2 than from the island, and, consequently, the effect of G2 on the trap is less than that on the island. Note that there is a current between these two boundaries, which indicates that the electron is not completely relaxed to the ground state of the island after the detrapping (see section I in the Supplementary Information). To achieve high-accuracy SE pumping, the operating point should be far from these boundaries.

To show further evidence of the detrapping process, we plot d*I*_P_/d*V*_UG_ as a function of *V*_UG_ and *V*_ENT_ at 6.5 GHz in Fig. 1(d). In this map, the boundary determined by the detrapping has a positive slope^24^ because, when *V*_UG_ is increased, *V*_ENT_ also needs to be increased in order to keep the electric field at the trap level constant [Fig. 1(f)]. In contrast, the ejection boundary has a negative slope because, when *V*_UG_ is increased, *V*_ENT_ needs to be decreased to keep the island potential constant [Fig. 1(g)] so that the tunnel rate through the exit barrier does not change. Since one of the boundaries between *ef* and 0 is the detrapping one, we conclude that the *ef* plateau corresponds to trap-mediated SE pumping. Note that we observe an additional boundary (white dashed line); the current drops to zero at the left side of the boundary because charge carriers in the Si wire (source and drain leads) are not induced due to insufficient *V*_UG_^32^.

### Nanoampere SE pumping at 7.4 GHz

**Figure 2:**
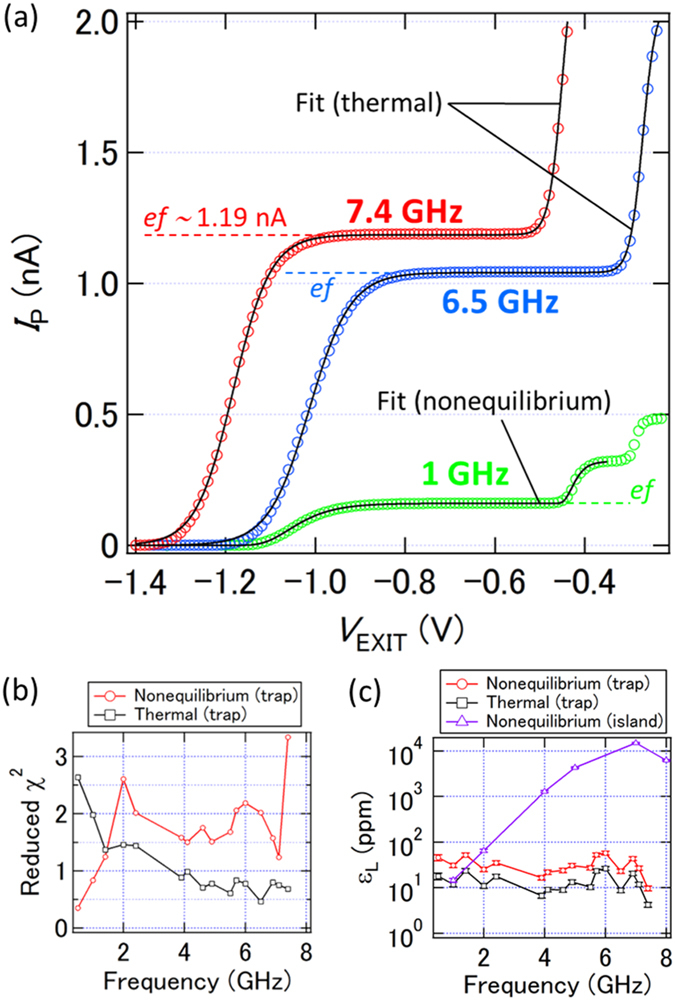
(**a**) *I*_P_ as a function of *V*_EXIT_ at 1, 6.5, and 7.4 GHz, where *V*_S_ = 0 V and *P* = 14 dBm. (*V*_ENT_, *V*_UG_) = (−1, 1), (−0.75, 1), and (−0.925, 0.95) V at 1, 6.5, and 7.4 GHz, respectively. Black lines are fits to the pumping characteristics using Eq. (1) (nonequilibrium) or Eq. (2) (thermal). (**b**,**c**) Reduced-*χ*^2^ values (**b**) and the lower bound of relative error rates at the inflection point of the *ef* plateau, *ε*_L_, (**c**) extracted from two types of fits to the pumping characteristics as a function of *f* (red circles and black squares). The error bar of each data point in the pumping characteristics is set to be 1%, which is a typical value. Purple triangles in (**c**) indicate *ε*_L_ extracted from a fit to a pumping current generated by another device, where a charge island defined by gate voltages is used (see section II in the Supplementary Information). Note that data points at around 3 GHz are missing because the pumping characteristics are strongly disturbed, probably due to a crosstalk effect of the high-frequency signal.

Figure 2(a) shows *I*_P_ as a function of *V*_EXIT_ at 1 (green circles), 6.5 (blue circles), and 7.4 (red circles) GHz, where we select *V*_ENT_ such that the current rise is determined only by the escape process: with reference to Fig. 1(c), the line of constant *V*_ENT_ intersects the escape boundary, but not the other three boundaries. Here, we again use the ammeter to record *I*_P_. The current level at the *ef* plateau at 7.4 GHz is about 1.19 nA, which is a record high current for a well-defined *ef* plateau.

To analyze the shape of the current plateau, we use a nonequilibrium electron capture model, known as the decay cascade model^16^,^30^, because the trap-mediated pump with an escape process as shown in Fig. 1(b) can be basically described within the same framework of dynamical electron capture. We use the decay cascade model with different proportionality coefficient to analyze *ef* (trap level) and 2*ef* (island) plateaus because the location of the trap level is different from that of the island. In this case, the equation is as follows:


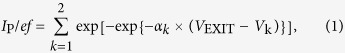


where *α*_1_ and *α*_2_ are proportionality coefficients for the trap level and island, respectively, and *V*_k_ is the threshold voltage of the *k*th current plateau. In addition, it is valuable to also fit the characteristics using an equation based on the generalized grand canonical distribution (thermal equilibrium limit)^16^,^33^,^34^:


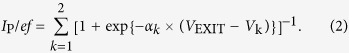


We fit the pumping characteristics by using these two equations and extract the reduced-*χ*^2^ values as a function of *f* [Fig. 2(b)]. We observe a crossover of the reduced-*χ*^2^ values at 1.5 GHz. The black lines in Fig. 2(a) are ones obtained by a fitting that generates a smaller reduced-*χ*^2^ value. Then, using the extracted parameters from the fit, we estimate *ε*_L_, which is a theoretical lower bound of a relative pumping error rate at the inflection point of the *ef* plateau^17^,^34^ [Fig. 2(c)]. For comparison, we add *ε*_L_ estimated from the island-mediated pumping in a different device (see section II in the Supplementary Information). While *ε*_L_’s for the island-mediated pumping increase dramatically with *f, ε*_L_’s for the trap-mediated pumping are almost independent of *f*. This is one of the most noteworthy properties of the single-trap electron pump.

### High-accuracy measurements of nanoampere pumping current

**Figure 3:**
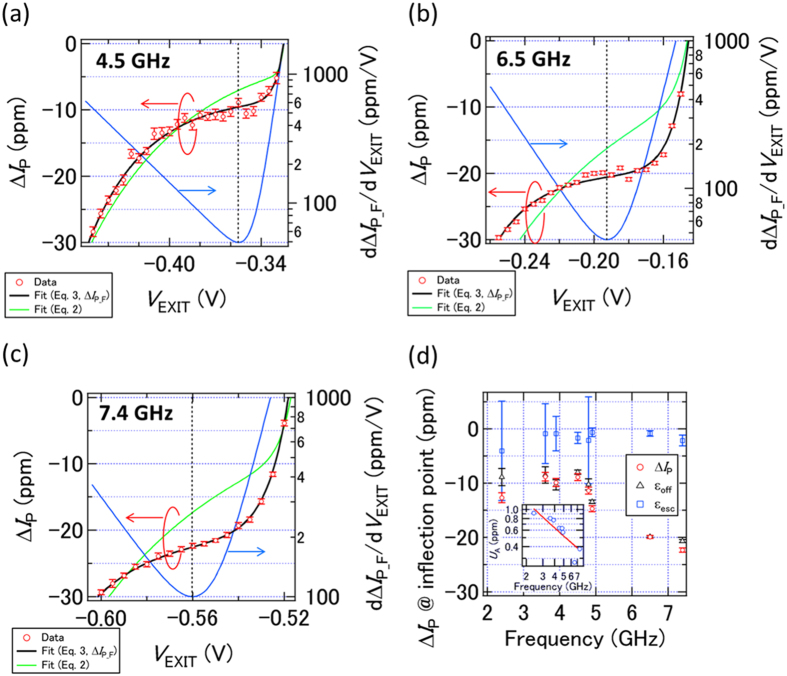
(**a**–**c**) Δ*I*_P_ = (*I*_P_ − *ef*)/*ef* (red circles, left axis), fitting curves to Δ*I*_P_ using Eq. 2 (green curves, left axis) and Eq. 3 (back curves, left axis), and the first derivative of the black curves with respect to *V*_EXIT_ (blue curves, right axis) as a function of *V*_EXIT_ at 4.5 (**a**), 6.5 (**b**), and 7.4 (**c**) GHz, where *V*_S_ = 0 V and *P* = 14 dBm. At 4.5 GHz, the temperature monitored by a thermometer in the cryostat *T* = 1.56 K, *V*_UG_ = 1 V, and *V*_ENT_ = −0.96 V. At 6.5 GHz, *T* = 1.34 K, *V*_UG_ = 0.975 V, and *V*_ENT_ = −1.05 V. At 7.4 GHz, *T* = 1.38 K, *V*_UG_ = 0.925 V, and *V*_ENT_ = −1.025 V. The error bar of Δ*I*_P_ indicates the type-A uncertainty. The black dashed line indicates *V*_EXIT_ at the inflection point of the black fit line. Integration time of each data point is about 13 min. (**d**) Δ*I*_P_ (red circles) at the *V*_EXIT_ value nearest to the inflection point as a function of frequency. The error bar of Δ*I*_P_ indicates the type-A uncertainty. Integration time of each data point is about 13 min. Black triangles and blue squares are the parameters *ε*_off_ and *ε*_esc_, respectively, extracted from fits to the high-resolution data using Eq. (3). The error bars for *ε*_off_ and *ε*_esc_ are larger for some frequencies because of a smaller number of measured points in these data sets. Inset: double logarithmic plot of the type-A uncertainty *U*_A_ shown in the main panel as a function of *f*. The red line is a fit to the data using an equation in which we assume that *U*_A_ is proportional to the inverse of *f*.

We next show measurement results of the trap-mediated pumping obtained using the high-accuracy measurement system with *I*_R_ tuned close to *ef*. Typical results at 4.5, 6.5, and 7.4 GHz are shown in Fig. 3(a–c), respectively (red circles). The vertical axis corresponds to the relative deviation of the current from an ideal value of *ef* [Δ*I*_P_ = (*I*_P_ − *ef*)/*ef*]. Here, the error bars of the individual data points indicate the random (type-A) uncertainty. While Eq. (2) is a good fit to the low-resolution data of Fig. 2(a), the fits to the high-resolution data using Eq. (2) [green lines in Fig. 3(a–c)] clearly deviate from the data. In order to take into account the error processes (such as electron loading errors or small gate leakage) that are not captured in the model used for Eq. (2), we add a constant offset term *ε*_off_ to Eq. (2) as follows:


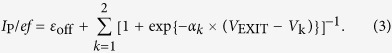


We fit the high-resolution data using Eq. (3) [black lines in Fig. 3(a–c)]. The improved fitting curves (Δ*I*_P_F_) agree well with the data at all frequencies, which indicates the existence of a small error source independent of the gate voltage. We also show the first derivative of the fit lines (*d*Δ*I*_P_F_/*dV*_EXIT_) with the vertical dashed lines showing the inflection points where the derivative is minimum. Figure 3(d) shows Δ*I*_P_ at the *V*_EXIT_ value nearest to the inflection point as a function of *f* (red circles). In addition, we define an escape error *ε*_esc_ which is the error we would observe if the additional error process described by *ε*_off_ was not present. We extract *ε*_esc_ at the inflection point by subtracting *ε*_off_ from Δ*I*_P_F_ at the inflection point. In Fig. 3(d), we also plot *ε*_off_ (black triangles) and *ε*_esc_ (blue squares). The result demonstrates that the escape error of about a few ppm is almost independent of frequency, which is consistent with the weak frequency dependence of *ε*_L_, and furthermore the experimentally measured error in Δ*I*_P_ is dominated by the constant term. Note that there is only about a 20-ppm-level deviation at the inflection point even at 7.4 GHz. This value is two orders of magnitude better than the previously reported one^20^ at such a high frequency.

Here, we mention the advantage of the high-frequency operation. The inset of Fig. 3(d) shows the type-A relative uncertainties in the main panel with an integration time of about 13 min. As expected, the relative uncertainty decreases linearly in a double logarithmic plot as the frequency (and therefore the current) is increased. At 6.5 and 7.4 GHz, the uncertainty is about 0.3 ppm. To achieve one order of magnitude improvement (0.03 ppm) at these frequencies, we only need an integration time of about one day (13 × 100 min). However, at 2.4 GHz (leftmost data), the integration time for achieving 0.03 ppm is more than a week. This indicates that a current level more than 1 nA is practically desirable to achieve an accuracy better than 10^−7^, although the measurement system as well as the pump itself must be improved.

**Figure 4:**
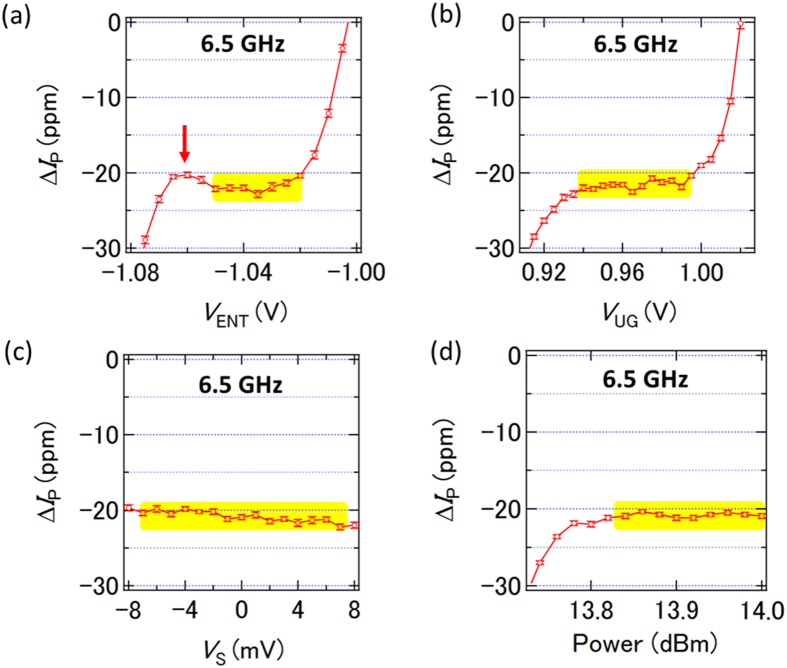
(**a**–**d**) Δ*I*_P_ as a function of *V*_ENT_ (**a**), *V*_UG_ (**b**), *V*_S_ (**c**), and *P* (**d**) at 6.5 GHz. In (**a**), *V*_UG_ = 0.975 V, *V*_EXIT_ = −0.19 V, *V*_S_ = 0 V, *P* = 14 dBm and *T* = 1.65 K. In (**b**), *V*_ENT_ = −1.05 V, *V*_EXIT_ = −0.19 V, *V*_S_ = 0 V, *P* = 14 dBm, and *T* = 1.36 K. In (**c**), *V*_UG_ = 0.975 V, *V*_ENT_ = −1.05 V, *V*_EXIT_ = −0.19 V, *P* = 14 dBm, and *T* = 1.98 K. In (**d**), *V*_UG_ = 0.975 V, *V*_ENT_ = −1.05 V, *V*_EXIT_ = −0.19 V, *V*_S_ = 0 V, and *T* = 1.34 K. The error bars indicate the type-A uncertainty. Integration time of each data point is about 13 min. Yellow regions indicate the current plateau. Red arrow indicates an anomalous current peak, which might be related to the crossover of the loading and escape processes.

For application as a metrological standard, robustness against changes to control voltages is an important consideration^35^. Figure 4(a–d) show the dependence of Δ*I*_P_ at 6.5 GHz as a function of *V*_ENT_, *V*_UG_, *V*_S_, and *P*, respectively. We can identify a current plateau (yellow regions) in all of these sweeps, and the current value on the plateau changes only by a few ppm, as is the case with the *V*_EXIT_ dependence in Fig. 3(b). This indicates that the pumping accuracy is maintained at a current level of more than 1 nA even when various voltage conditions are changed. Note that we observe a current peak as a function of *V*_ENT_ as indicated by a red arrow. The origin is not clear but it might be related to the crossover of the two transfer processes: the loading and escape processes.

## Discussion

Here we discuss possible origins of the weaker frequency dependence of *ε*_L_ for the trap-mediated pumping compared to the island-mediated one. At higher frequency, the number of captured electrons is determined at an earlier time during the pump cycle, with a correspondingly lower entrance barrier^15^. At this point, the junction capacitance across the entrance barrier is larger. When the dominant capacitance is due to the junction, the corresponding charging energy is smaller, resulting in accuracy degradation as a function of *f*. This is one possible cause of the accuracy degradation in the island-mediated pumping^15^. In contrast, since the trap level is well localized, it could be more isolated from the leads than the island is. In this case, the charging energy would be determined not by junction capacitance but by self-capacitance, which should be independent of the capture point in time.

Another possible reason for the accuracy degradation is related to nonadiabatic excitation^15^,^22^. When a change in the potential shape is fast during the rise of the entrance barrier, a single electron in the ground state is excited and spills out to the source lead. In the case of the trap level, the change in the potential shape should be slow because the gate-induced confinement is not the main contribution to the confinement of the trap level. In addition, the trap level may have a large gap between the ground and excited states because of the strong confinement. These points would lead to a small probability of excitation even at a high frequency.

An interesting observation is that the pumping mechanism changes from the nonequilibrium capture (asymmetric rise shape) to the thermal equilibrium one (symmetric rise shape) at 1.5 GHz shown in Fig. 2(b). A similar mechanism crossover was observed in a Si SE pump using a charge island^34^ owing to heating caused by the leakage of the power of the high-frequency signal, where *ε*_L_ increases with increasing heating due to thermal errors. In contrast, since *ε*_L_ is almost constant as a function of *f* in Fig. 2(c), it would not be reasonable to explain the crossover by the simple heating effect. One possible explanation is a quantum fluctuation^36^, which leads to a similar symmetric rise shape. This effect should become pronounced when the rise time of the barrier becomes fast. Nevertheless, we need further studies to elucidate the physical origin of the mechanism crossover.

The ~20-ppm pumping accuracy demonstrated at 7.4 GHz is a new benchmark result, but further improvement is required for the closure of the QMT. To discuss this, we estimate an effective charge addition energy *E*_add_ ~ 7.5 meV, defined by the energy difference between the trap level and the island (see section III in the Supplementary Information). One possible reason for this small value is that the trap level may not be deep enough in this specific device. Since *ε*_esc_ is related to the ratio of *E*_add_ to temperature, a device with a deeper trap level could reduce the error caused by the escape process. More importantly, we must address the problem of the gate-independent constant error source parameterized as *ε*_off_ in Eq. (3). Although the origin is not clear and further studies are necessary, we speculate that possibilities are an error in the electron loading process or small gate leakage. In future experiments, we need to evaluate a device with the desired depth, such as one with a deep trap level of about 37 meV^24^.

One point that we should discuss is the low device yield of the single-trap electron pump. As discussed in our previous report^24^, the problem could be circumvented by using dopants positioned in a controlled manner^1^,^26^ instead of trap levels. However, as long as one high-accuracy single-trap electron pump is found, we might be able to perform QMT experiments with high precision owing to the high-speed characteristics demonstrated in this letter. In that sense, the single-trap electron pump would be promising for closing the QMT.

In conclusion, we have demonstrated that a single-trap electron pump operating at 7.4 GHz generates a nanoampere current with a relative deviation of about 20 ppm from the exactly quantized value *ef*. This high accuracy at such a high pumping speed represents a significant improvement compared to previous results at *f* > 1 GHz. An important point for achieving the high-speed operation would be that the confinement of the trap level is strong and not electrically defined. These results represent an important step toward realizing the quantum current standard, especially for QMT experiments. In addition, this device architecture might be useful for quantum information processing using a trap level^37^.

## Methods

### Device fabrication

The Si wire is patterned using electron beam lithography, followed by the formation of a thermal oxide with a thickness of 30 nm. The wire thickness and width are 15 and 25 nm, respectively. Next, we form two lower gates (G1, G2), which are made of heavily doped n-type polycrystalline Si. The lower-gate length is 40 nm and the spacing between the two lower gates is 100 nm. Then, an inter-layer oxide with a thickness of 50 nm is formed using chemical vapor deposition. After that, we form an upper gate, which covers the entire region of the Si wire. Finally, n-type source and drain leads are formed using ion implantation with the upper gate used as a mask.

### Measurement system

The current measurement system depicted in Fig. 1(a) has been used in high-accuracy measurements of GaAs^17^,^18^ and Si^20^ SE pumps. In this system, we use a 1-GΩ standard resistor *R*, which is traceable to the quantum Hall resistance standard. By applying a voltage to *R*, we generate reference current *I*_R_. The applied voltage is measured by a voltmeter, which is directly calibrated by the Josephson voltage standard. From measurements of *I*_R_ and *I*_DIF_ (=*I*_R_ − *I*_P_), we can determine *I*_P_ with an uncertainty of about 1 ppm. The measurement details, including the uncertainty of the system, are described in ref. 20. The measurement temperature is 1–2 K in a helium-3 cryostat without condensation.

## Additional Information

**How to cite this article:** Yamahata, G. *et al*. High-accuracy current generation in the nanoampere regime from a silicon single-trap electron pump. *Sci. Rep.* **7**, 45137; doi: 10.1038/srep45137 (2017).

**Publisher's note:** Springer Nature remains neutral with regard to jurisdictional claims in published maps and institutional affiliations.

## Supplementary Material

Supplementary Information
