# Supplementary material for: High-accuracy current generation in the nanoampere regime from a silicon single-trap electron pump
Source: Sci Rep. 2017 Mar 21;7:45137. doi: 10.1038/srep45137 (PMC5359665; doi:10.1038/srep45137)
Supplement: Supplementary Information [file srep45137-s1.pdf]

Supplementary Information: High-accuracy current generation in a nanoampere regime from a silicon single-trap electron pump

Gento Yamahata,[1] Stephen P. Giblin,[2] Masaya Kataoka,[2] Takeshi Karasawa,[1] and Akira Fujiwara[1]
[1] *NTT Basic Research Laboratories, NTT Corporation, 3-1 Morinosato Wakamiya, Atsugi, Kanagawa 243-0198, Japan*
[2] *National Physical Laboratory, Hampton Road, Teddington, Middlesex TW11 0LW, United Kingdom*

Relaxation process

We discuss the relaxation process after the detrapping in more detail. Figure S1(a) shows the same current map as that in Fig. 1(c) in the main text. At the red circle in Fig. S1(a), a detrapped electron can be ejected to the drain even when the relaxation to the ground state of the island occurs as shown in the potential diagram in Fig. S1(b), because of a sufficiently large ejection probability. At the blue square in Fig. S1(a), a detrapped electron can be ejected to the drain without relaxation, but when the relaxation of the detrapped electron occurs, the electron is captured by the island as shown in the potential diagram in Fig. S1(c). This is because the exit barrier is higher than the island ground state energy. At the green triangle in Fig. S1(a), since the detrapping is prohibited, there is no current flow even if the ejection probability is high for a hot electron as shown in the potential diagram in Fig. S1(d).

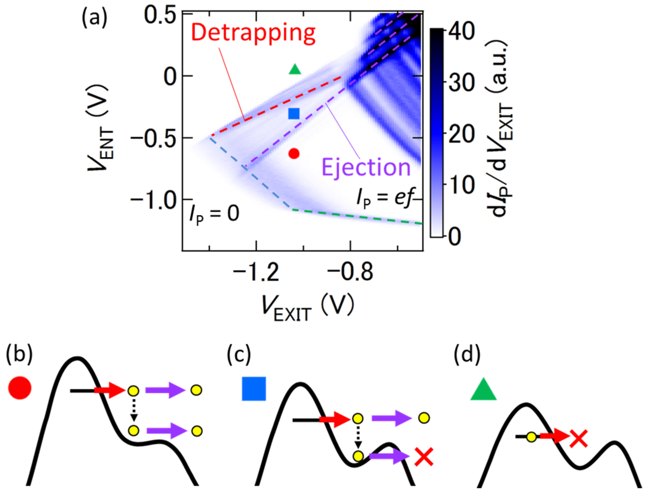

Figure S1. (a) Current map, which is the same as that in Fig. 1(c) in the main text. (b)-(d) Electron potential diagrams at the red circle (b), blue square (c), and green triangle (d) in (a). The red and purple arrows correspond to the detrapping and ejection process. The dashed arrow corresponds to the relaxation process.

Error estimation of island-mediated SE pumping

We used another device to investigate the tunable-barrier SE pump with an electrically defined charge island. The structure is almost the same as the device in the main text. The only difference is the width of the Si wire, which is about 10 nm. Figure S2(a) shows dI_P/dV_{EXIT} as a function of V_{EXIT} and V_{ENT}. Here, we do not observe the detrapping process explained in the main text, which indicates that the SE is pumped via the island between G1 and G2. Figure S2(b) shows the pumping characteristics at 1 and 8 GHz as a function of V_{EXIT}. We fit I_P using Eq. (1) in the main text with a condition of $\alpha_1 = \alpha_2$ [black lines in Fig. S2(b)]. ϵ_L's between 1 and 8 GHz are estimated from the fitting results [Fig. 2(c) in the main text]. Note that 8 GHz is the highest frequency among the published pumping data; it yields a current level of about 1.28 nA, although ϵ_L is about 10^{-2}.

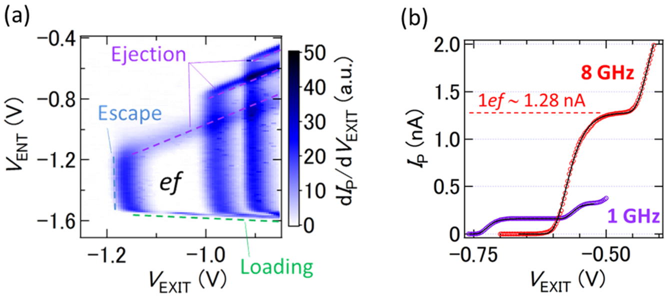

Figure S2. (a) First derivative of I_P with respect to V_{EXIT} as a function of V_{EXIT} and V_{ENT} at 50 MHz, where $V_{UG} = 3$ V, $V_S = 0$ V, and $P = 9$ dBm. The measurement temperature T is 4.2 K in liquid He. (b) I_P as a function of V_{EXIT} at 1 (purple circles) and 8 (red circles) GHz, where $V_{UG} = 1.5$ V, $V_S = 0$ V, and $P = 10$ dBm. $V_{ENT} = -1.2$ and -0.6 V at 1 and 8 GHz, respectively. Black lines are fits to the pumping characteristics. The error bar of each data point in the pumping characteristics is set to be 1 %, which is a typical value. T is 4.2 K in liquid He.

Estimation of effective electron addition energy

Figure S3(a) shows a schematic of the electron potential diagram during the rise of the entrance barrier

with the definition of an effective electron addition energy $E_{\rm add}$. Here, we assume that one electron is dynamically captured by the island with a probability of about half and another electron is captured by the trap level. In other words, the voltage condition corresponding to this diagram is at a current rise between ef and $2ef$ plateaus. Figure S3(b) shows $I_{\rm P}$ of the trap-mediated SE pumping in the device shown in the main text as a function of $V_{\rm UG}$ at 1 (blue circles) and 17 (red circles) K, where $I_{\rm P}$ is normalized by ef. Since the plateau width is smaller at 17 K than at 1 K, the escape process shown in Fig. 1(b) in the main text depends on the temperature and it should be dominated by thermal hopping at 17 K[1]. To estimate $E_{\rm add}$, we fit the characteristics at 17 K using Eq. (1) in the main text. For simplicity, we assume that $V_{\rm UG}$ does not modulate the entrance barrier top. This would be reasonable because of the screening of the $V_{\rm UG}$ effect by G1. In this case, $E_{\rm add} = \alpha_1 kT(V_2 - V_1)$, and it is estimated to be about 7.5 meV from the extracted fitting parameters.

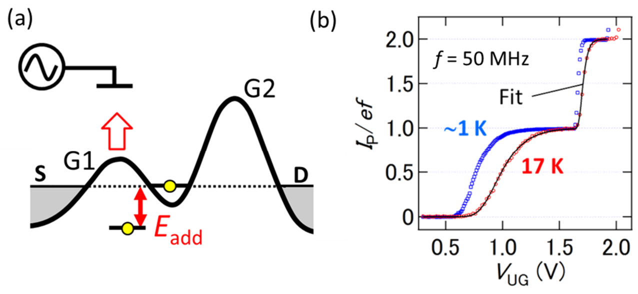

Figure S3. (a) Electron potential diagram with the definition of the effective electron addition energy $E_{\rm add}$. (b) $I_{\rm P}$, which is normalized by ef, as a function of $V_{\rm EXIT}$ at 1 and 17 K at 50 MHz. At 1 K, $V_{\rm EXIT} = -0.72$ V, $V_{\rm ENT} = -1$ V, $V_{\rm S} = 0$ V, and $P = 8$ dBm. At 17 K, we applied voltage pulses to the entrance gate, where the on-state and off-state voltages are 0 and -3 V, which roughly corresponds to $P \sim 8$ dBm and $V_{\rm ENT} = -1.5$ V, $V_{\rm EXIT} = -1$ V, and $V_{\rm S} = 0$ V. The black line is a fit to the pumping characteristics.

[1] Yamahata, G., Karasawa, T. & Fujiwara, A. Gigahertz single-hole transfer in Si tunable-barrier pumps. *Appl. Phys. Lett.* **106**, 023112 (2015).
